# Insights into the Phylogeny of *Ustilago maydis* Strains via Comparative Analysis of Their Respective Mitogenomes

**DOI:** 10.3390/jof12030206

**Published:** 2026-03-13

**Authors:** Dennis Doe, Anthony Vu, Joseph P. Ham, Michael H. Perlin

**Affiliations:** Department of Biology, University of Louisville, Louisville, KY 40208, USA; dennis.doe@louisville.edu (D.D.); anthony.vu@louisville.edu (A.V.); joseph.ham@louisville.edu (J.P.H.)

**Keywords:** mitotype, *Ustilago maydis*, mitochondria, biotroph

## Abstract

*Ustilago maydis* is an economically significant biotrophic smut fungus, capable of infecting maize. This is a localized infection where tumors are formed, potentially in any of the aboveground parts of the plant. In extreme cases, maize plants may die. It is also dimorphic, i.e., it is capable of switching from yeast-like to filamentous forms. The switch can be induced by nitrogen sources, pH, and some lipids/oils. The active infectious form is the filamentous form which is capable of penetrating plant cells using the appressorium. This study focuses on understanding the mitochondrial genome diversity in *U. maydis*, the selection pressure on the genes encoded in the mitochondrial genome, and the phylogeny of the strains investigated. The results suggest that the strains maintained high consistency in genome architecture and synteny. The *cox1* and *cob* genes in the genomes possessed different intron numbers, with the presence or absence of homing endonuclease genes (HEGs), which overall contributed to the differences in the genome sizes. Among the genes in the mitogenome, *nad6* was the only gene that has a non-synonymous nucleotide change, but the overall changes within the mitogenomes suggest purifying selection. The study helped identify the different mitotypes using PCR, although further markers or whole-genome sequencing may be required to fully distinguish mitotypes.

## 1. Introduction

Since its introduction by Holliday for recombination studies [1], *Ustilago maydis* has emerged as an important system for a variety molecular genetics studies, and importantly, as a model for fungal/plant interactions. *U. maydis* is a biotrophic smut fungus. Unlike necrotrophs, biotrophic fungi like *U. maydis* require a live host plant to complete its lifecycle. As part of its lifecycle, the fungus causes gall or tumor formation on all aboveground parts of host plants, i.e., maize or its ancestor, teosinte. Infection of a host is preceded by the mating of compatible yeast-like haploid cells, known as sporidia. Successful mating yields a dikaryotic filamentous and infectious growth form that leads to the formation of appressorium, a structure that helps the fungus penetrate the cell walls of host plants [2]. This process relies on cues from the host surface to facilitate stable mating and carbon sources to complete their lifecycle. *U. maydis* produces diploid teliospores at the end of a successful infection. When these teliospores are released from the galls, and are transmitted to a suitable surface, they germinate, meiotic segregation occurs, and haploid cells are produced from subsequent budding of probasidia [3].

As discussed above, compatible mating by cells of opposite mating-type allows cigar-shaped yeast-like sporidial cells to transition to dikaryotic filamentous hyphae. Another means for sporidia to filament is when starved for nitrogen [4]. Moreover, filamentation can be triggered by growth in low-pH media [5] or via exposure to lipids, including corn oil [6]. *U. maydis* is a convenient model system that has allowed examination of a variety of fundamental concepts in biology, like genetic recombination, as well as important revelations concerning fungal plant parasites and pathogens, e.g., how plant pathogens invade host tissues without triggering host defense response [7]. A highly developed molecular genetic toolkit has been developed for *U. maydis* and its close relatives (e.g., *Sporisorium reilianum*). The haploid sporidial form produces discrete colonies on agar media; it is easily transformed for gene disruption or over-expression studies; and it also bears a relatively smaller genome size of ~20 Mb. The smaller genome size facilitates comparative genomics approaches. *U. maydis* haploid strains are morphologically similar, making it hard to distinguish individual strains based on appearance alone. Mitochondrial genes can serve as molecular markers for differentiating strains within the same species [8]. Studies that focused on identifying mitotypes in *U. maydis* usually used genes like the ribosomal large subunit (LSU) [9]. This gene provides a basis of differentiation but limited information into the complex diversity in the mitochondria genome of *U. maydis*. For instance, intron invasions and other genetic events in the mitochondrial genome may have occurred during evolution, and such changes are unlikely to be revealed using solely on LSU polymorphisms. Therefore, there is the need to fully characterize the mitochondrial genomes of the various mitotypes that have been described [9].

Mitochondria are crucial organelles particularly in obligate aerobes, where they are necessary for survival. In some species, i.e., petite mutants in *S. cerevisiae*, the cells can survive without a functional mitochondrion [10]. The survival of most eukaryotic cells depends on the mitochondria. The dysfunction of these organelles is associated with aging, diseases and other biological processes [11,12]. Endosymbiotic theory explains the origin of the mitochondria and this organelle is always termed “the powerhouse of the cell”. The electron transport chain (ETC) known to produce most of the ATP needed for the cells is embedded in the inner membrane of the mitochondria. The mitochondria have their own genetic material (mtDNA) which replicates independently of the nuclear genome and is found in the mitochondrial matrix. The vast amount of information and features in the mitochondrial genome facilitates studies in population genomics, taxonomy, and their uniparental inheritance (UPI) [9,13,14,15]. The mitogenome is known to undergo low levels of recombination, making it possible for tracing the lineage and evolution of species specifically in animals [16]. The mitogenomes in basidiomycete fungi have not been studied as much as in humans and yeast cells. The limited information available suggests that the mitogenomes of most species are circular [17], with a few linear ones [18]. The size of mitochondrial genomes ranges from about 11 kb to about 343 kb [19], signifying the diversity of the mitogenome in the fungal clade. A standard mitochondrial genome possesses 14 protein coding genes (PCGs) (*atp9*, *atp6*, *atp8*, *nad1-6*, *nad 4L*, *cox1*, *cox2*, *cox3*, *cob*) as well as genes encoding a ribosomal protein s3 (*rsp3*), ribosomal RNAs (*rnl* and *rns*), all of which are known to be highly conserved in the mitogenomes in the fungal clade [20]. tRNAs are known to be sandwiched between these conserved genes. The genomes also contain different numbers of introns in different genes. Intron studies in the mitogenome reveal high invasion of introns, which further contributes to the size variations [21,22]. Within the introns are encoded homing endonuclease (HEGs) which have self-splicing capabilities. These HEGs are thought to facilitate the invasion or mobility of introns within the mitogenome [13,23]. To date, only one mitogenome of *U. maydis* is available (NCBI Reference Sequence: NC_008368.1). This limits our understanding of the complexity of the *U. maydis* mitochondrial genomes and possibly why some mitochondria may be preferred to others in terms of their inheritance.

In this study, the genomes of 14 haploid strains of *U. maydis* were sequenced and assembled. The following questions were addressed in this study: 1. What is the extent of diversity in the mitogenome of *U. maydis* and which genes vary between the strains? 2. Are any of the mitochondrial genes undergoing selection pressure? 3. Are there any reliable mitochondrial markers that would allow us to distinguish one mitotype from the other? 4. What evolutionary mechanisms drive the current mitochondrial architecture in *U. maydis*? The mitogenomes of these 14 haploid strains were annotated for gene features, i.e., gene content, tRNAs, base composition, PCGs, synteny, phylogeny, etc. The mitogenomes of these individual strains were compared. To the best of our knowledge, this is the first time the whole mitogenomes of different strains has been accomplished in *U. maydis*. The comparative mitogenomic data will also provide insight for evolutionary studies of proteins and genes within basidiomycetes, as well as providing different molecular markers for mitogenomic studies, particularly investigations of mitochondrial inheritance.

## 2. Materials and Methods

Strains and growth conditions. The *U. maydis* strains used in this study were obtained from J. Kämper and C. Basse [9] and are listed in Table 1, including mating type and mitotypes.

Haploid strains were grown in yeast peptone sucrose (YPS) broth on a rotary shaker at 220 rpm at 28 °C or on solid potato dextrose (PD) agar at the same temperature. For long-term storage, strains were maintained in YP-glycerol (15%) media and kept at −80 °C or on PD agar at 4 °C for no longer than 7 days.

Whole-Genome Isolation, Sequencing and Assembly. The whole-genome isolation, sequencing and assembly were performed by Plasmidsaurus Limited (Louisville, KY, USA) using Oxford Nanopore long-read sequencing. Genomic DNA was prepared and sequenced using the Oxford Nanopore Ligation Sequencing Kit (v14 chemistry) with sequence-independent tagmentation to minimize fragmentation. Libraries were sequenced on R10.4.1 flow cells using a primer-free protocol. Raw sequencing data in FASTQ format was basecalled using Dorado with the super-accurate basecalling model and Q10 quality filtering.

Quality filtering was performed using Filtlong v0.2.1, removing the lowest 5% of reads with heavy weight applied to low-quality reads (-qual-weight 10). Genome assembly was performed using Hifiasm v0.25.0 with parameters optimized for high-quality Oxford Nanopore reads. The assembled genome achieved approximately Q50-60 accuracy (99.999–99.9999%, corresponding to one error per 100,000–1,000,000 bases).

Genome annotation was performed using Augustus v3.5.0 to predict genes with the best model automatically selected based on the closest reference genome. Open reading frames were aligned against the Uniprot database (v2024_04) using BLAST v2.15.0, with the top hit retained if e-value < 0.05. Assembly quality was assessed using BUSCO v5.7.1 for genome completeness and contamination, and Bandage v0.8.1 for contig visualization and analysis (Plasmidsaurus, Louisville, KY, USA). The assembly was validated by blastn analysis against NCBI database. The presence of the 14 PCGs was used as a further verification of the sequences obtained. The ORFs were also investigated for the appropriate start and stop codons for all the PCGs. MFannot (http://megasun.bch.umontreal.ca/cgi-bin/mfannot/mfannotInterface.pl) (accessed on 6 March 2025) and Mitos2 (https://usegalaxy.org/?tool_id=toolshed.g2.bx.psu.edu%2Frepos%2Fiuc%2Fmitos2%2Fmitos2%2F2.1.9%2Bgalaxy0&version=latest) (accessed on 12 March 2025) were used to annotate the genomes; tRNA finder on galaxy [24] was used to identify and annotate the tRNAs. Any uncertainties in the sequences were further resolved through blastp analysis on NCBI using the predicted amino acids. Additionally, gene annotations of predicted proteins were confirmed by comparing with homologs from other species using blastp against the NCBI database.

### Molecular and Bioinformatics Analysis

Mega 12 software [25] was used to align mitotypes and for phylogeny analysis. NCBI ORF finder (https://www.ncbi.nlm.nih.gov/orffinder/) (accessed on 12 June 2025) was used to identify the ORFs for the different mitotypes. Primers were designed to distinguish the various polymorphic/distinct regions amongst the mitotypes and also to identify non-synonymous nucleotide changes within the genome for Nad6. Primer combinations and PCR conditions for each have been documented in Table S2. Mitochondrial maps were drawn with OGDRAW [26].

To analyze the selection pressure on each gene, MUSCLE (on MEGA 12) was used to align the nucleotide sequences. The alignments were then used to calculate the dN and dS values for each gene. Likelihood ratio tests were used to identify the best-fitting model for all these analyses.

Phylogeny. Whole mitochondrial sequences for the 14 strains were used to infer the phylogeny. They were aligned using MUSCLE (MEGA 12 software) and the best model was first checked. The best evolutionary fit-model was with the Tamura 3-parameter with evolutionary-invariant (T92 + I) and with 1000 bootstraps.

## 3. Results

### 3.1. General Features of Mitogenomes

The general characteristics of the mitogenomes of 14 strains were analyzed and are provided in Table 2. There were 22 tRNAs encoded for GF5, GF8, GF25 and GF63, with the remaining strains having 23 each. The missing tRNA for the former group was that specifying cysteine. tRNAs for methionine and leucine had two copies each across all strains. Serine tRNA was encoded by two gene copies in GF5, GF8, GF25 and GF63, with the remaining strains having three copies each. The two copies of the encoded tRNAs for methionine (CAT) and leucine (TAG) were the same, with serine having two copies of the same tRNA (GCT) gene and one copy for another anti-codon (TGA) combination. In the strains with 22 tRNAs, they were missing an extra copy of GCT tRNA for serine. The tRNA size ranges were from 70 to 88 bp.

### 3.2. Gene Order Among the Strains

**Figure 1:**
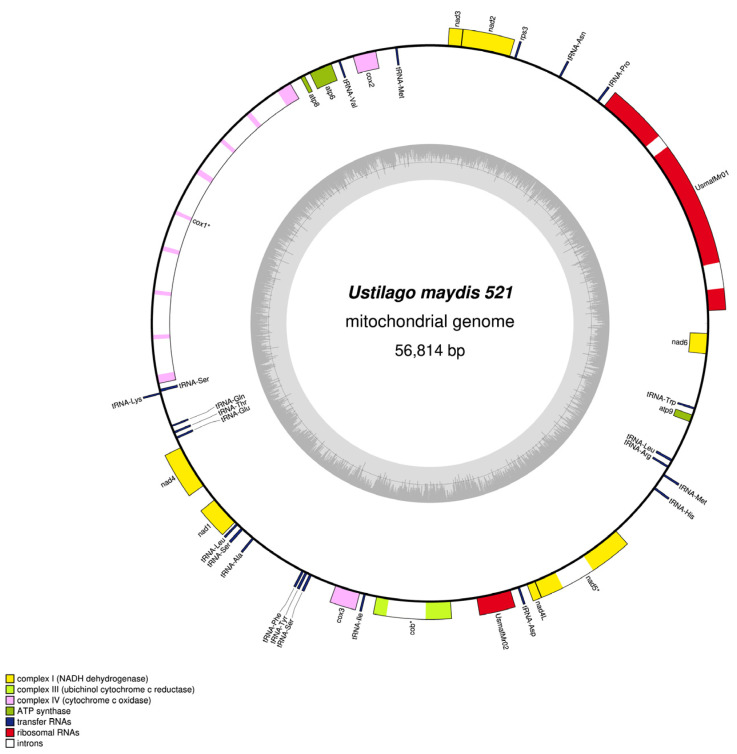
Circular map of the mitogenome of *U. maydis* 521 strain. Genes are represented by different colored blocks. Colored blocks outside each ring indicate that the genes are on the direct strand, while colored blocks within the ring indicate that the genes are located on the reverse strand. The mitogenome maps of the other strains are present in the Supplementary Data, Figure S1. Genes with asterisk indicates intron-containing genes.

The synteny analysis showed a high conservation of the PCGs in the mitogenome of *U. maydis*, maintaining gene order and arrangement throughout the mitogenomes (Figure 1, Figures S1 and S2).

These genes were tightly organized, from *cox1*, *atp8*, *atp6*, *cox2*, *nad3*, *nad2*, *nad6*, *atp9*, *nad5*, *nad4L*, *cob*, *cox3*, *nad1*, and *nad4*, with minimal structural variations between the strains. Within the PCGs, genes encoding rRNAs and tRNAs are sandwiched between them, maintaining their relative positions and orientations. This demonstrates remarkable stability in mitochondrial genome organization across these strains of *U. maydis*.

### 3.3. Codon Usage/Preference in the Mitogenomes

**Figure 2:**
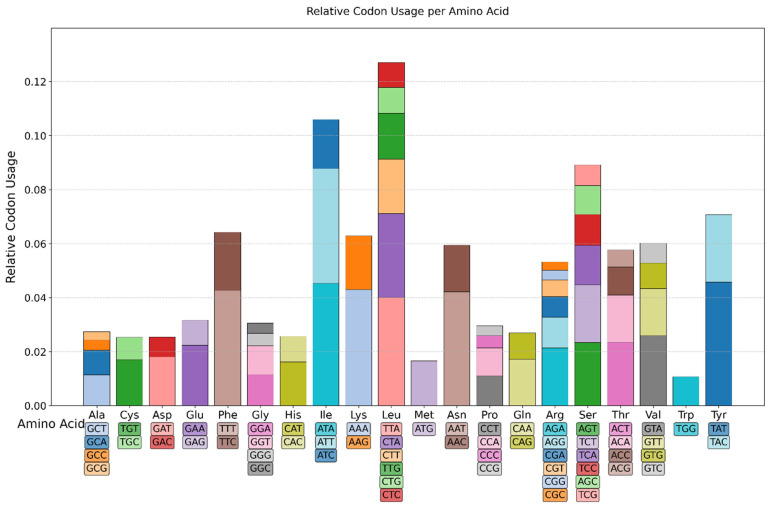
Codon usage/preference in the 14 strains analyzed shows high preference for hydrophobic amino acids. Different color patterns represent the different codon combinations for the different amino acids present in the mitogenome. Relative frequency of occurrence/usage is plotted on the y-axis and amino acids on the x-axis.

The codon usage frequencies across the 14 PCGs were assessed by concatenating all the PCGs, and this was then used for the calculations. Leucine showed the highest number of occurrences or usage in the genes analyzed, followed by isoleucine, with tryptophan having a low occurrence rate (Figure 2).

The *cox1* gene has an alternate start codon which is 72 bp before the canonical start position. All the PCGs begin with the canonical start codon (ATG) and end with different stop codons. For instance, *nad5* has TAG as the stop codon, but the other remaining genes have TAA.

### 3.4. Intron Numbers Shape the Genomic Architecture in U. maydis cox1 and cob Genes

**Figure 3:**
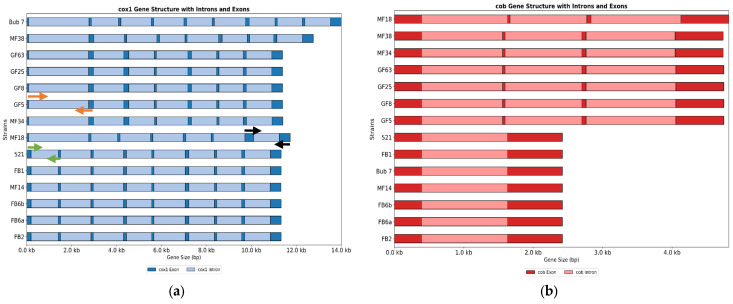
*cox1* (**a**) and *cob* (**b**) genes have different exon–intron numbers and high variability in the exon and intron distributions. Dark colors represent exons and lighter ones, introns. Arrows indicate the position and direction of diagnostic primers on reference genomes (**a**). Black arrows indicate primer set 1; green, set 2; and orange, set 3 (Tables S1 and S2). Mitotypes have been grouped into Group A (521, FB1, FB2, FB6A, FB6B, and MF14), group B (BUB7), C(MF18), D (MF34, GF5, GF8, GF25, GF63) and E (MF38).

Analysis of the cytochrome c oxidase subunit 1 (*cox1*) and the apocytochrome b (*cob*) genes among the 14 mitogenomes in this study revealed a variety of exon–intron arrangements. The *cox* gene has a maximum of nine introns in BUB7, whereas GF5, GF8, GF25, MF18, and MF34 each have seven introns, and the remaining strains (MF38, 521, FB1, FB2, FB6a, FB6b and 521) have eight introns each. The sizes of the exon and the introns varied across the strains for *cox1* except for the last exon in all the strains, which was highly conserved (Figure 3a).

Similar observations were made for the *cob* gene. The 521, FB1, FB2, FB6a, FB6b, and BUB7 orthologues had only two exons. The remaining strains had four exons in their respective *cob1* genes. In this gene, the sequence for exon 1 was highly conserved across strains. The size differences in the *cob* genes for the different strains were a result of the number of intron insertions (Figure 3b). The respective primers designed to distinguish mitotypes yield varying PCR product sizes as listed in Tables S1 and S2.

### 3.5. Selection Pressure on Mitogenomes

#### 3.5.1. The Mitochondrial Genes Exhibit Different Selection Pressures

**Figure 4:**
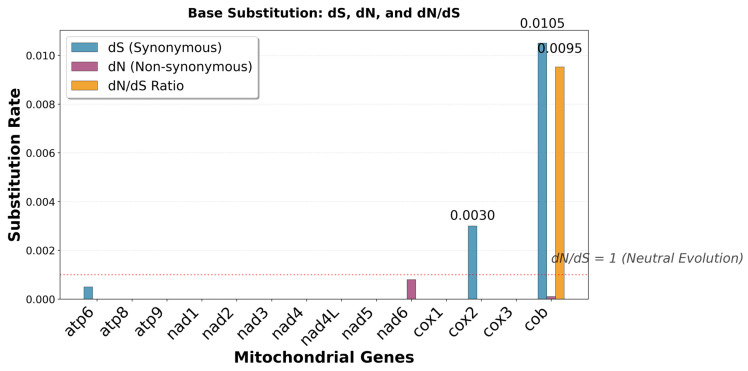
Genetic analysis of 14 protein coding genes conserved in mitogenomes of *U. maydis*. K2P, the Kimura-2-parameter distance used for the analysis; dS, the mean synonymous substitution rate; dN, the mean non-synonymous substitution; dN/dS, the ratio of non-synonymous to synonymous substitution. All analyses were carried out with MEGA 12 [25].

The selection pressure on each of the genes was assessed by calculating the synonymous and non-synonymous substitution rates for the 14 PCGs using MEGA 12. The apocytochrome b (*cob*) gene showed the highest level of substitutions in terms of synonymous and non-synonymous base changes. *atp8*, *atp9*, *nad1*, *nad3*, *nad4L*, *cox1*, *cox3*, *nad4*, and *nad5* genes had no base changes. *cox2* and *atp6* showed a low level of synonymous changes. The overall dN/dS value of all the genes was below 1, which indicates the PCGs are under purifying selection (Figure 4).

#### 3.5.2. Nucleotide Change in *nad6*

**Figure 5:**
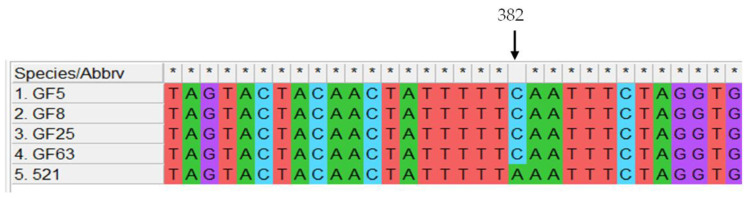
The non-synonymous change within the nad6 gene. The comparison was made with the 521 mitogenome. The change was at position 382 in its protein coding sequence. This polymorphism was confirmed by PCR amplification from the genomic region of the GF strains of the region predicted to contain the change, and subsequent Sanger sequencing. Asterisk indicates identical sequences.

*nad6* had a non-synonymous nucleotide change at position 382. The nucleotide change resulted in an amino acid change from Lysine (K) to Glutamine (Q), that is from AAA to CAA (Figure 5).

The alpha fold analysis of protein structure [27] suggests that the change may not necessarily affect protein function and structure as the amino acid change existed in a fold which might not necessarily be the active site of this enzyme (Figure S3).

### 3.6. Phylogeny Tree of U. maydis

**Figure 6:**
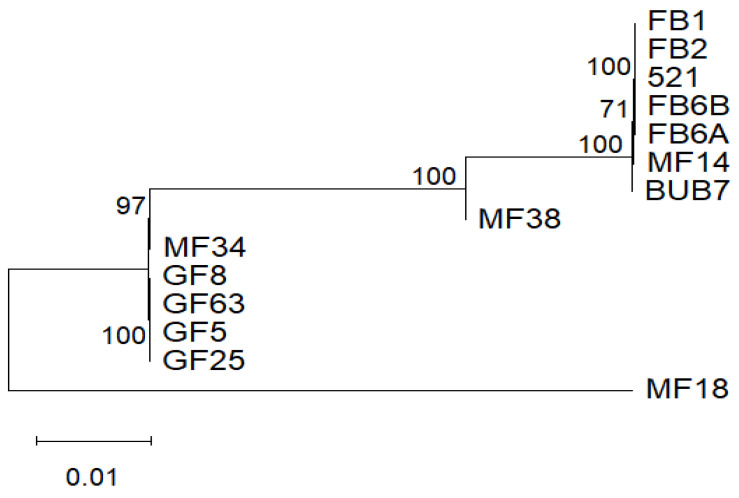
The phylogenetic tree was calculated from multiple sequence alignment of the combined mitochondrial gene set (14 PCGs) of the 14 strains used. Phylogeny was inferred using the Tamura 3-parameter with the maximum likelihood option. The phylogenetic tree was drawn using MEGA 12 software [25].

The maximum likelihood method was used. The best evolutionary fit-model was with the Tamura 3-parameter with evolutionary-invariant (T92 + I) with 1000 bootstraps performed. The 14 PCGs were concatenated and aligned for the phylogeny analysis using MEGA 12. The phylogeny showed different evolutionary times for each of the strains. The percentage probability of occurrence of the branches in the phylogeny was above average except for the branching between BUB7 and MF14, with 71% support (Figure 6). Another tree using the *Sporisorium reilianum* SRZ2 mitogenome [28] as an outgroup (Figure S4) was also generated. In this analysis, the *U. maydis* mitotypes broke into two groups, the first with 97% support (521, FB1, FB2, FB6a, FB6b, MF14 and Bub7) and the second with 68% support (MF18, MF38, MF34, GF8, GF63, GF5, and GF25).

### 3.7. Intron Analysis Shows “Gain and Loss Mechanism” Through Evolutionary Time

**Figure 7:**
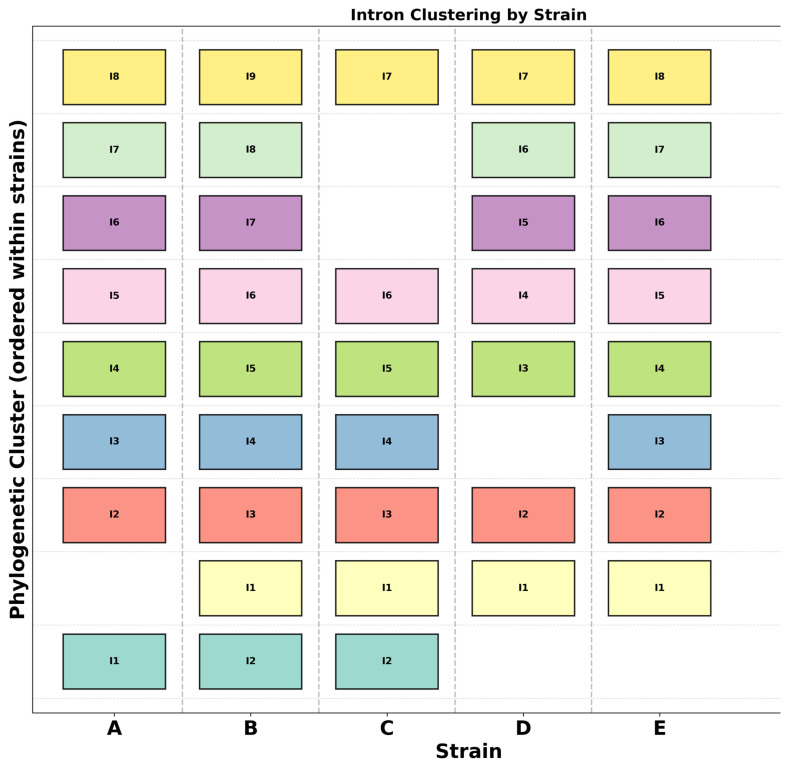
Intron analysis was carried out to assess the relationship between the introns in the *cox1* gene. Each block represents the position of each intron in the specific category of strains. Blocks with the same color represent similarities (>99%) between the strains. A (521, FB1, FB2, FB6A, FB6B, MF14), B (BUB7), C (MF18), D (MF34, GF5, GF8, GF25, GF63) and E (MF38).

Intron similarities and differences were assessed based on their sequences. The sequences from the introns within each cluster were aligned using MEGA 12 and their characteristics based on phylogeny analysis were inferred. Blocks depicting the same color share higher sequence similarities and conservation. For instance, the last introns were highly conserved across the strains analyzed based on position (Figure 7).

The number of introns varies from strain to strain possibly due to loss and gain of introns. The HEG domains of the introns were removed before alignment as their presence may interfere with alignment. HEGs may be acquired under some conditions and may not be representative of the introns present.

### 3.8. Proposed Method of Identifying the Different Mitotypes

**Figure 8:**
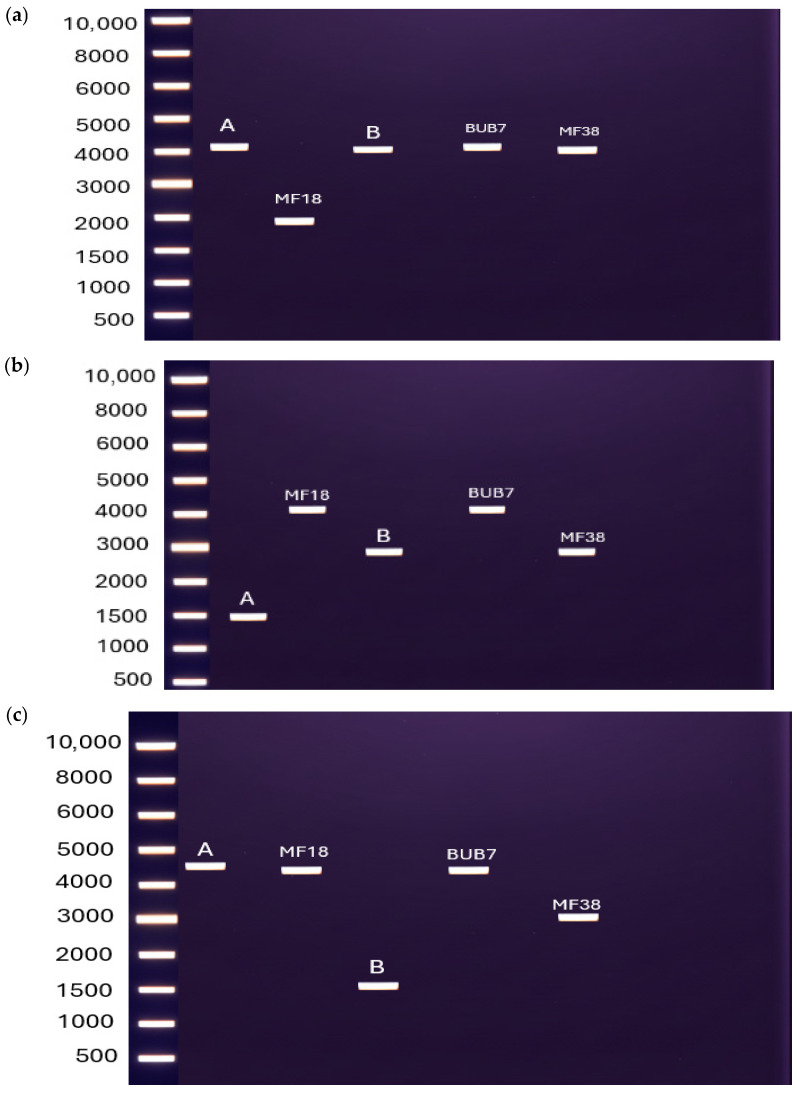
The schematic agarose gels show the various band sizes based on the different primer sets used in Table S1. Band A is produced for strains 521, FB1, FB2, FB6A, FB6B and MF14; band B corresponds to GF5, GF8, GF25, GF63 and MF34. The primers were able to distinguish some individual strains like MF18 (with primer set 1), MF38 (with primer set 3) and BUB7 (combinations of primer sets 1 and 2). The schematic image is a compiled image after PCRs. The actual gels can be found in the Supplementary Figure (Figure S5). All primers and combinations are listed in Table S1. Gel representation from primer set 1 (**a**), set 2 (**b**) and set 3 (**c**).

We were interested in using the identified mitotype differences to develop diagnostic PCR approaches. Figure 8 describes the differences between amplification sizes of *cox1* genes which was later used to distinguish mitotypes for mitochondrial inheritance studies. The primers used are discussed in Table S1 and the expected sizes for amplification documented in Table S2. The positions of the primers used are shown in Figure 3.

## 4. Discussion

This study focuses on characterizing a collection of different mitochondrial genomes in *U. maydis*. Mitochondrial genomes offer a great opportunity for species and possibly strain characterization and identification [29,30,31]. Different strains within the same species clade have been shown to exhibit different mitochondrial genome sizes [32], and the *U. maydis* strains analyzed were no exception. In *U. maydis*, the largest mitogenome was from MF38 with 62,461 bp and the smallest were GF5, GF8, GF25, GF63 and MF34 with 56,085 bp. This shows variability in the mitogenomes in *U. maydis*, mainly in terms of the genome sizes. Introns have been shown to be the major driver of mitogenome size variations as reported in other studies [33,34]. The major mitogenome size difference appeared to be due to variations in the *cox1* and *cob* gene sizes (Figure 3). These genes have a variable number of introns, which made a major contribution to the size differences of the mitogenomes among the strains analyzed, similar to what has been reported in *Sporisorium reilianum* [28]. Introns are known to harbor selfish elements like the LAGLIDADG and the GIY-YIG endonucleases. These are enzymes that facilitate self-splicing and intron invasion [23,35,36,37]. The observation of different intron positions within the genome may indicate intron invasions through gain-and-loss, as the gene sequences and lengths were highly conserved. Gain and loss of introns shape genome architecture [38], and were assessed here in the *cox1* gene. The analysis (Figure 7) was based on whether a specific intron appeared in three or more mitotype clusters but was missing in any. Cluster B (BUB7) appears to retain all the introns available in *cox1*. Cluster A was used as a reference, as this group includes strain 521, the well-established reference mitogenome available on NCBI. Apart from introns I2, I4, I5 and I8, which appear in all the mitotypes, the remaining five introns were not fully conserved across the different mitotypes, at the position level. Some clusters appear to have lost some of the introns. For instance, introns I6 and I7 were missing in cluster C, intron I3 was missing in cluster D, and intron I1 was missing in clusters D and E (Figure 7). This mechanism affected the exon distributions in the *cox1* gene. Some exons appeared to have been split into two exons from a single exon (exon 1 and 2 in all clusters except A), while others appear to have come together after losing an intron (exon 7 in cluster C) (Figure 3a and Figure 7). These gaps illustrated in the figure support that the gain and loss of introns shape the architecture of the genome. The results confirm the hypothesis of intron-poor species undergoing more intron-loss-and-gain events, as the Ustilagomycotina subdivision taxa are known to house the least introns in their mitogenomes compared with other members of the basidiomycete division [39]. The substantial variation in intron number and position between the *cox1* and *cob* genes across the strains provides substantial evidence of evolutionary divergence within the species clades examined. BUB7 has the most introns (9), with the GF strains and MF18 having seven each in the *cox1* genes; surprisingly, the last exons for all the strains were highly conserved in terms of their position. This suggests that although the gene itself is undergoing evolutionary pressure in terms of intron insertions, the last exon may encode essential portions of the core enzyme structure and must be conserved to maintain function. Fungal genomic analysis suggests that intron gain and loss normally occur at the beginning of genes rather than at the end. This may also explain why the last exon has been conserved [39].

In fungal systems, particularly in the basidiomycete phylum, the number of PCGs are highly conserved [20]. The arrangement of these PCGs may be highly conserved in some species [40], but less conserved overall in the basidiomycete phylum [40]. In the strains analyzed, the gene order was highly conserved. The tight organization of the protein-coding genes (PCGs), ribosomal RNAs, ribosomal protein subunit 3 (rps3) and tRNAs may be an indication of functional constraints operating on the mitochondrial genome architecture and possibly help with reducing or accumulating mutations, as the mitochondria normally lack a DNA damage repair system [41]. This observation suggests high mitogenome stability in *U. maydis*, although the intron–exon combinations in the *cox1* and *cob* genes are clearly exceptions. This together with the high AT content (~68%), which is typical with fungal mitogenomes [34,42,43], reflect different evolutionary pressures on the genome architecture, PCGs and genome sizes. Surprisingly, RNase P, which is absent in all basidiomycetes mitogenomes [44,45], was found to be encoded in the mitogenomes of the strains analyzed.

The nucleotide substitutions observed for the PCGs together with the dN/dS (Figure 4) indicate that some of the genes were undergoing purifying selection changes. Most of the substitutions resulted in codons that had tRNAs encoded in the mitogenome. For instance, phenylalanine appeared to be the amino acid with most changes in the gene(s) encoding its tRNAs, TTT-TTC (Table S3), with the latter codon being the one with the tRNA present in the mitogenome. This change has much evolutionary significance, as energy requirements may be dissimilar when the cells switch from yeast-like to filamentous growth. Therefore, purifying selection may enhance energy production, as translation will be more efficient with the tRNA present in the mitochondria for genes required for oxidative phosphorylation [46]. Contrary to the assertion that intron-less genes undergo positive selection on their protein coding genes, the *cob1* genes in *U. maydis* showed the highest positive selection on its protein coding sequence [38]. This suggests that compensatory adaptation is required for tRNA demands even on unexpected sequences, like the *cob* gene. MF38 seems to have undergone significantly more changes compared to other strains. There is a possibility that this gene is under evolutionary pressures not acting on the other mitogenes. The non-synonymous change at position 382 in the GF-clade strains warrants further investigation for functional changes or protein stability, although protein structure was unaltered (Figure S2). The changes to meet the tRNA requirements for the purifying selection, particularly for the *cob* gene, would be an adaptive evolutionary mechanism to meet such requirements.

The tRNA gene number of the respective mitogenomes ranged from 22 to 23, with those for methionine and leucine having two copies each, serine having three copies each in some, but others having just two copies. Strains with 22 tRNA genes lack an extra copy of serine (GCT) tRNA (Table 2). The extra copy of the serine (GCT) tRNA in some strains suggests duplication through evolutionary time. A total of 19 amino acid tRNAs were encoded within the mitogenome, with the exception being that for cysteine, which was missing. The canonical mitochondrial genetic codon usually requires all standard tRNAs, yet the absence of cysteine tRNA could be due to tRNA importation from the cytoplasm as reported in yeast and other organisms [47,48,49]. In the codon usage chart (Figure 3), all the different codon combinations for various amino acids were present, suggesting possible tRNA import, as earlier reported [50,51]. The tRNA size range from about 70 to 88 bp across all strains is consistent with maintenance of functional constraints on tRNA structure, likely related to ribosomal binding or aminoacylation efficiency [52,53].

Mitogenomic analysis has widely been adopted in population genetics and evolutionary studies [44,53]. *U. maydis* strains are difficult to distinguish morphologically between subspecies or strains, due to similar morphological traits. In some studies, single genes, for instance, rRNAs and ITS regions, have been used to infer phylogeny [54,55]. In *U. maydis*, the protein coding genes are highly conserved, offering little information for phylogenic analysis. Therefore, whole mitogenome or PCG phylogenies may provide reliable phylogenies as more genetic information is used and may be supported statistically [56,57,58,59]. The whole mitogenome phylogeny provided a well-supported phylogeny as it was able to distinguish the mitotypes. The high bootstrap support values indicate confidence in branching relationships, except for the clade BUB7-MF14 showing about 71% probability. This may reflect an incomplete lineage sorting or recent divergence. Clustering at various nodes supports the various clusters of the mitotypes identified through genome size and gene structure analysis, suggesting that mitochondrial genome divergence is a reliable indicator of evolutionary relationships at the population level. The identification of distinct evolutionary times for each strain implies *U. maydis* has undergone significant diversification with differential rates of molecular evolution across lineages. This may reflect different effective population sizes, geographic isolation or varying generation times among strains.

The proposed molecular markers for mitotype identification, based on PCR amplification of the *cox1* gene with variable intron–exon structure, demonstrates a promising approach for rapid strain differentiation. The ability to distinguish several individual strains, MF18, MF38 and BUB7, using specific primer combinations, coupled with the ability to assign other strains to defined groups, provides a practical framework for strain identification without full genome sequencing; the variation in PCR product sizes correlating with intron number and position validates the hypothesis that intron–exon structure is the primary source of size polymorphism in the amplicons [34].

However, the current approach does not distinguish strains within the two large groups (A and B). This limitation reflects that such strains have not fully diverged from each other and suggests additional molecular markers or whole-genome approaches may be necessary for the complete strain resolution. However, PCR approaches as described in this report may be useful in characterizing mitochondrial inheritance patterns in crosses between different mitotype parental strains.

## Figures and Tables

**Table 1:** List of strains used in this study, including mating type and mitotypes.

Strain	Genotypes	Mitotypes ^a^
521 ^b^	a1b1	F
FB1 ^b^	a1b1	F
FB2 ^b^	a2b2	F
FB6a	a2b1	F
FB6b	a1b2	F
MF14 ^c^	a2b4	F
MF18 ^c^	a1b17	W
MF34 ^c^	a1b14	W
GF5 ^c^	a2b13	W
GF8 ^c^	a2b13	W
GF25 ^c^	a1b13	W
GF63 ^c^	a1b13	W
BUB7 ^d^	a1b3	B
MF38 ^c^	a1b18	B

^a^ Modified from [9]. The letters in the Mitotypes column (F, W, and B) are the designations used in [9] to represent the various groups identified by these authors using RFLPs, with a focus on differences within the LSU rRNA region. ^b^ Originally isolated by R. Holliday, near St. Paul, Minnesota, USA; FB1, FB2, FB6a, and FB6b are from the same teliospore from the cross of 518 and 521. ^c^ Isolated as meiotic segregants from teliospores collected from the Marburg area, Germany, between 2004 and 2006. ^d^ Isolated as meiotic segregants from teliospores collected in the Bonn (Germany) area.

**Table 2:** Comparison of the different mitotypes.

Strain	Length (bp)	AT%	GC%	tRNAs	Introns
FB2	56,814	68.80%	31.20%	23	14
GF5	56,085	68.00%	31.99%	22	12
GF8	56,085	68.00%	31.99%	22	12
GF25	56,085	68.00%	31.99%	22	12
GF63	56,085	68.00%	31.99%	22	12
BUB7	61,449	68.18%	31.83%	23	16
FB6B	56,814	68.80%	31.20%	23	14
MF38	62,431	68.15%	31.85%	23	17
MF34	56,087	68.01%	31.99%	23	12
MF18	56,460	68.11%	31.89%	23	12
MF14	56,803	68.80%	31.20%	23	14
FB1	56,814	68.80%	31.20%	23	14
FB6A	56,814	68.80%	31.20%	23	14
521	56,814	68.80%	31.20%	23	14

The average AT and GC contents for the strains were about 68% and 32%, respectively, demonstrating a high AT content for these mitogenomes. The smallest mitogenome size sequenced was 56,807 bp, which was observed in GF5, GF8, GF25, and GF63, while MF38 had the largest mitogenome with 62,431 bp. The 14 PCGs were present in the mitogenomes together with the ribosomal protein subunit 3 (rps3). Rase P was present in all the mitogenomes of the strains analyzed.

## Data Availability

All the sequences used in this study are available upon request. The mitogenome for 521 is available on NCBI under the accession number NC_008368.

## Supplementary Materials

The following supporting information can be downloaded at https://www.mdpi.com/article/10.3390/jof12030206/s1, Figure S1. Circular maps of the mitogenomes of the different mitotypes; Figure S2. Gene order among the strains; Figure S3. Nad6 protein structure using AlphaFold; Figure S4. Phylogeny of mitogenomes with *S. reilianum* as an outgroup; Figure S5. Gel images from mitotype determination; Table S1: Primer sets with their annealing temperatures; Table S2: Expected band sizes for distinguishing mitotypes; Table S3: Nucleotide substitutions within the PCGs.

## Abbreviations

The following abbreviations are used in this manuscript:

PCGs	Protein Coding Genes
LSU	Ribosomal Large Subunit
HEGs	Homing Endonuclease Genes
PCR	Polymerase Chain Reaction
ETC	Electron Transport Chain
mtDNA	Mitochondrial DNA
UPI	Uniparental Inheritance
