# Supplementary material for: Insights into the Phylogeny of Ustilago maydis Strains via Comparative Analysis of Their Respective Mitogenomes
Source: J Fungi (Basel). 2026 Mar 13;12(3):206. doi: 10.3390/jof12030206 (PMC13028006; doi:10.3390/jof12030206)
Supplement: Supplementary file 1 [file jof-12-00206-s001.zip › jof-4184639-supplementary.pdf]

Supplementary information for mitochondria data.

SUPPLEMENTARY DATA.

Table S1. Primer sets with their respective annealing temperatures.

Name		Sequence	Annealing Temperature
Set 1	Forward	TCCCAGGATTTGGTATGGTT	55
	Reverse	TGGAATGGTGTTGGTGATTC	
Set 2	Forward	TGGAACAGCATTTTCAGTGC	55
	Reverse	CCTGCCCCTTGTTCTACAAA	
Set 3	Forward	CAGCTATGGTCGGTGGATTC	55
	Reverse	AGTGTCATTCCTGGGTTTCG	
Nad 6	Forward	CGGTGCTGTTCTTTCTGGAAT	53
	Reverse	TGTGTATCCGCAGCAAAAGT	

All the reactions had an extension time of 5 minutes to allow completion of larger fragment amplification.

Table S2. Expected band sizes for distinguishing mitotypes based on the various primers used.

	521	FB1	FB2	FB6a	FB6b	MF14	MF18	MF34	MF38	GF5	GF8	GF25	GF63	BUB7
Set 1	4208	4208	4208	4208	4208	4208	1952	4158	4150	4158	4158	4158	4158	4208
Set 2	1500	1500	1500	1500	1500	1500	4144	2954	2954	2954	2954	2954	2954	4181
Set 3	4358	4358	4358	4358	4358	4358	4202	1670	3018	1670	1670	1670	1670	4225

The numbers in green represent the reference genome that was used to design each primer set.

Table S3. Nucleotide substitutions within the PCGs of *U. maydis* strains.

	PCGs			
STRAIN	COB	COX2	NAD6	ATP6
MF14		T—A (360)		
MF18	T—A (435), A—T (438, 474), T—C (483)			T—C (663)
MF34	T—C (387, 483), T—A (435), A—T (438, 474)	T—G (435)		
MF38	T—C (387, 408, 483), A—T (405, 438, 474), T—A (387, 435)	T—G (435)		
GF5	T—C (387, 483), T—A (435), A—T (438, 474)	T—G (435)	A—C (382)	
GF8	T—C (387, 483), T—A (435), A—T (438, 474)	T—G (435)	A—C (382)	
GF25	T—C (387, 483), T—A (435), A—T (438, 474)	T—G (435)	A—C (382)	
GF63	T—C (387, 483), T—A (435), A—T (438, 474)	T—G (435)	A—C (382)	

The PCGs were compared to the reference genome (521) available on NCBI (https://www.ncbi.nlm.nih.gov/nucleotide/NC_008368.1) and was determined to be identical to the re-sequenced version of the mitogenome determined for this study. With reference to Figure 4, the genes that showed some synonymous and/or non-synonymous substitutions were selected and the individual changes within the PCGs were assessed. Within the PCGs, the *cob1* gene showed the most single nucleotide changes (SNPs), with *cox2, nad6,* and *atp6* also showing some changes. The MF38 mitogenome appears to have acquired a larger number of nucleotide substitutions in these genes compared with the other mitotypes.

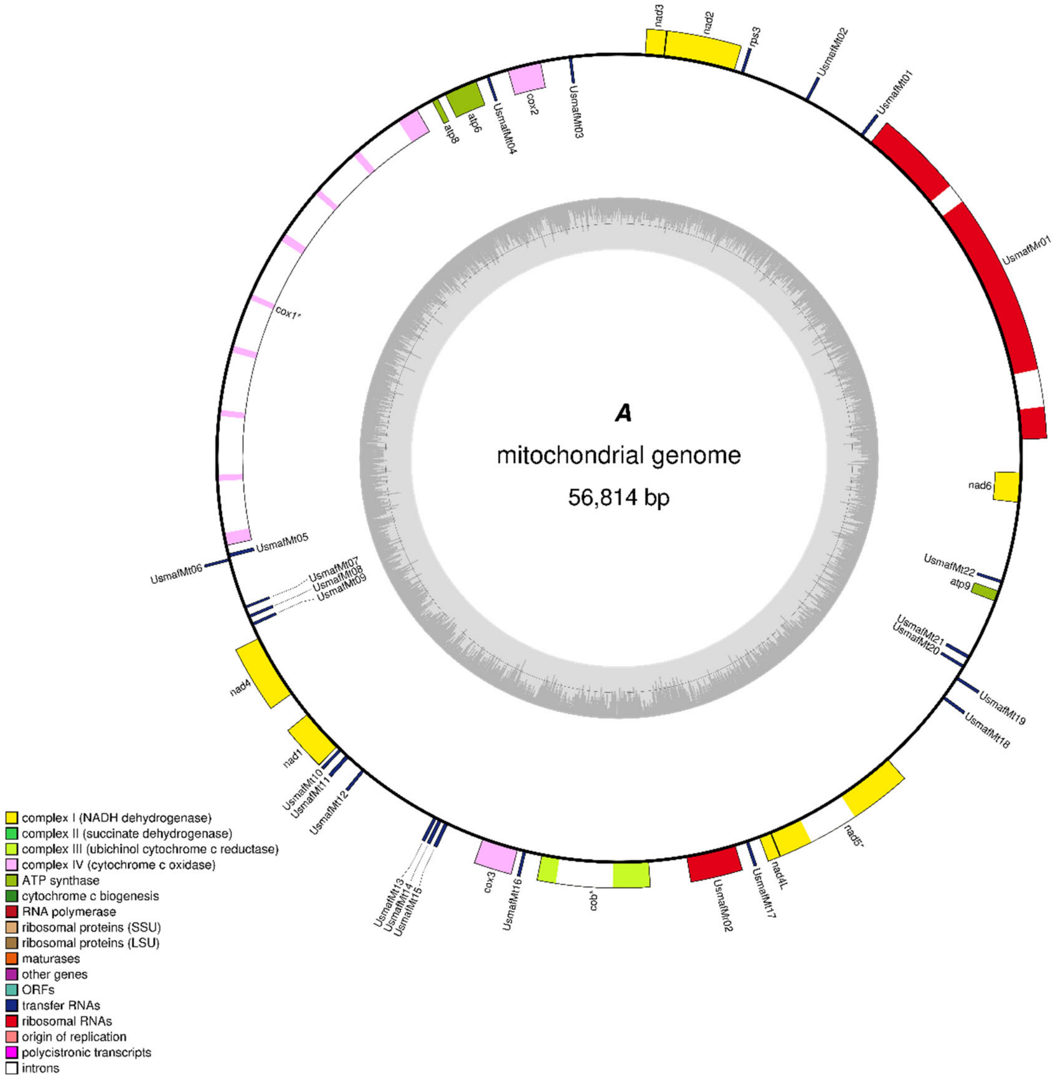
A
mitochondrial genome
56,814 bp
nad3
nad2
rps3
UsmafMt02
UsmafMt01
UsmafMr01
nad6
UsmafMt22
atp9
UsmafMt21
UsmafMt20
UsmafMt19
UsmafMt18
nad5*
nad4L
UsmafMt17
UsmafMr02
cob*
UsmafMt16
cox3
UsmafMt13
UsmafMt14
UsmafMt15
UsmafMt12
UsmafMt11
UsmafMt10
nad1
nad4
UsmafMt07
UsmafMt08
UsmafMt09
UsmafMt06
UsmafMt05
cox1*
atp8
atp6
UsmafMt04
cox2
UsmafMt03
complex I (NADH dehydrogenase)
complex II (succinate dehydrogenase)
complex III (ubichinol cytochrome c reductase)
complex IV (cytochrome c oxidase)
ATP synthase
cytochrome c biogenesis
RNA polymerase
ribosomal proteins (SSU)
ribosomal proteins (LSU)
maturases
other genes
ORFs
transfer RNAs
ribosomal RNAs
origin of replication
polycistronic transcripts
introns

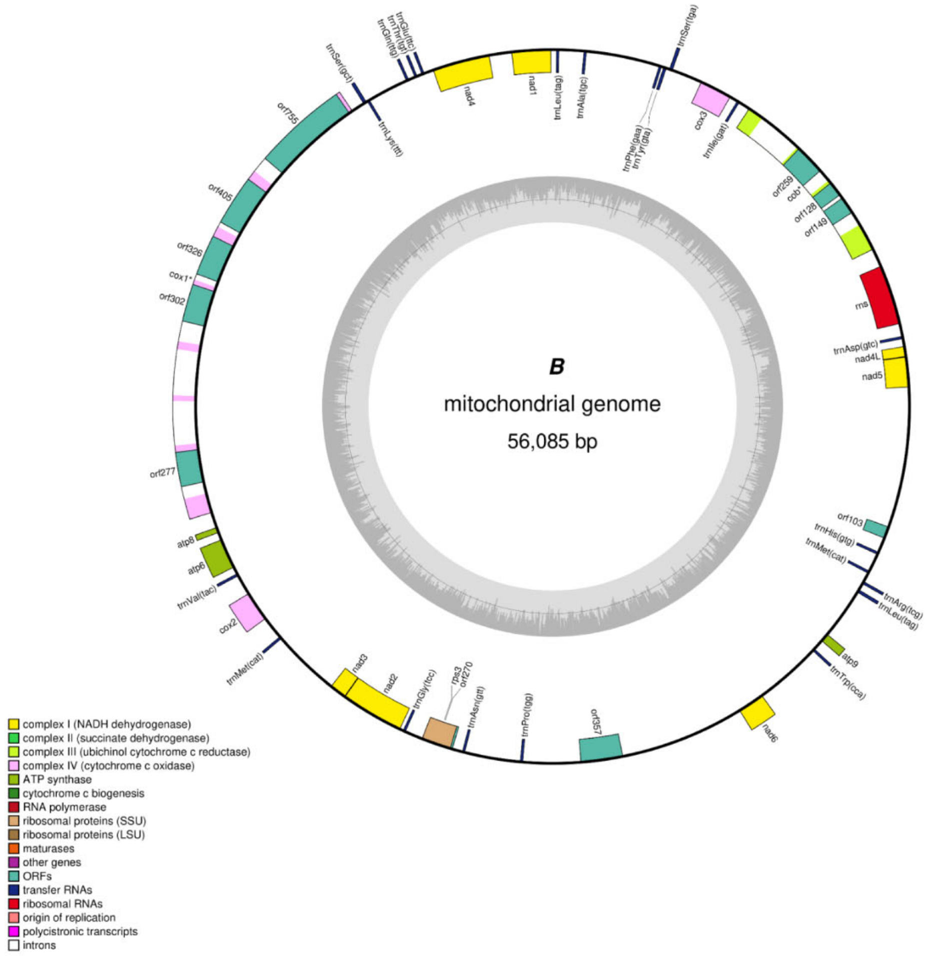
B
mitochondrial genome
56,085 bp
nad4
nad1
trnLeu(tag)
trnAla(tgc)
trnSer(tga)
trnPhe(gaa)
trnTyr(gta)
cox3
trnIle(gat)
orf259
cob*
orf126
orf149
rns
trnAsp(gtc)
nad4L
nad5
orf103
trnHis(gtg)
trnMet(cat)
trnArg(tcg)
trnLeu(tag)
atp9
trnTrp(cca)
nad6
orf357
trnPro(tgg)
trnAsn(gtt)
rps3
orf270
trnGly(tcc)
nad2
nad3
trnMet(cat)
cox2
trnVal(tac)
atp6
atp8
orf277
orf302
cox1*
orf326
orf405
orf755
trnSer(gct)
trnLys(ttt)
trnGln(ttg)
trnThr(tgt)
trnGlu(ttc)
complex I (NADH dehydrogenase)
complex II (succinate dehydrogenase)
complex III (ubichinol cytochrome c reductase)
complex IV (cytochrome c oxidase)
ATP synthase
cytochrome c biogenesis
RNA polymerase
ribosomal proteins (SSU)
ribosomal proteins (LSU)
maturases
other genes
ORFs
transfer RNAs
ribosomal RNAs
origin of replication
polycistronic transcripts
introns

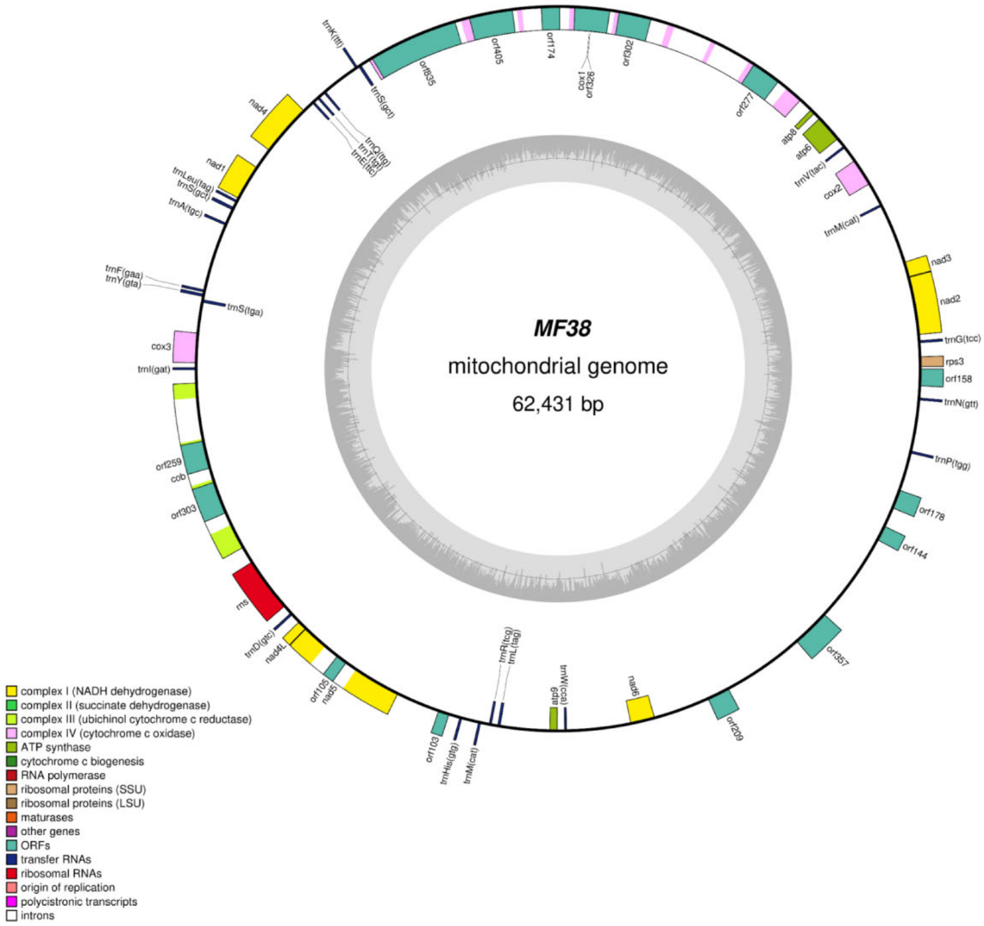
MF38
mitochondrial genome
62,431 bp
complex I (NADH dehydrogenase)
complex II (succinate dehydrogenase)
complex III (ubichinol cytochrome c reductase)
complex IV (cytochrome c oxidase)
ATP synthase
cytochrome c biogenesis
RNA polymerase
ribosomal proteins (SSU)
ribosomal proteins (LSU)
maturases
other genes
ORFs
transfer RNAs
ribosomal RNAs
origin of replication
polycistronic transcripts
introns

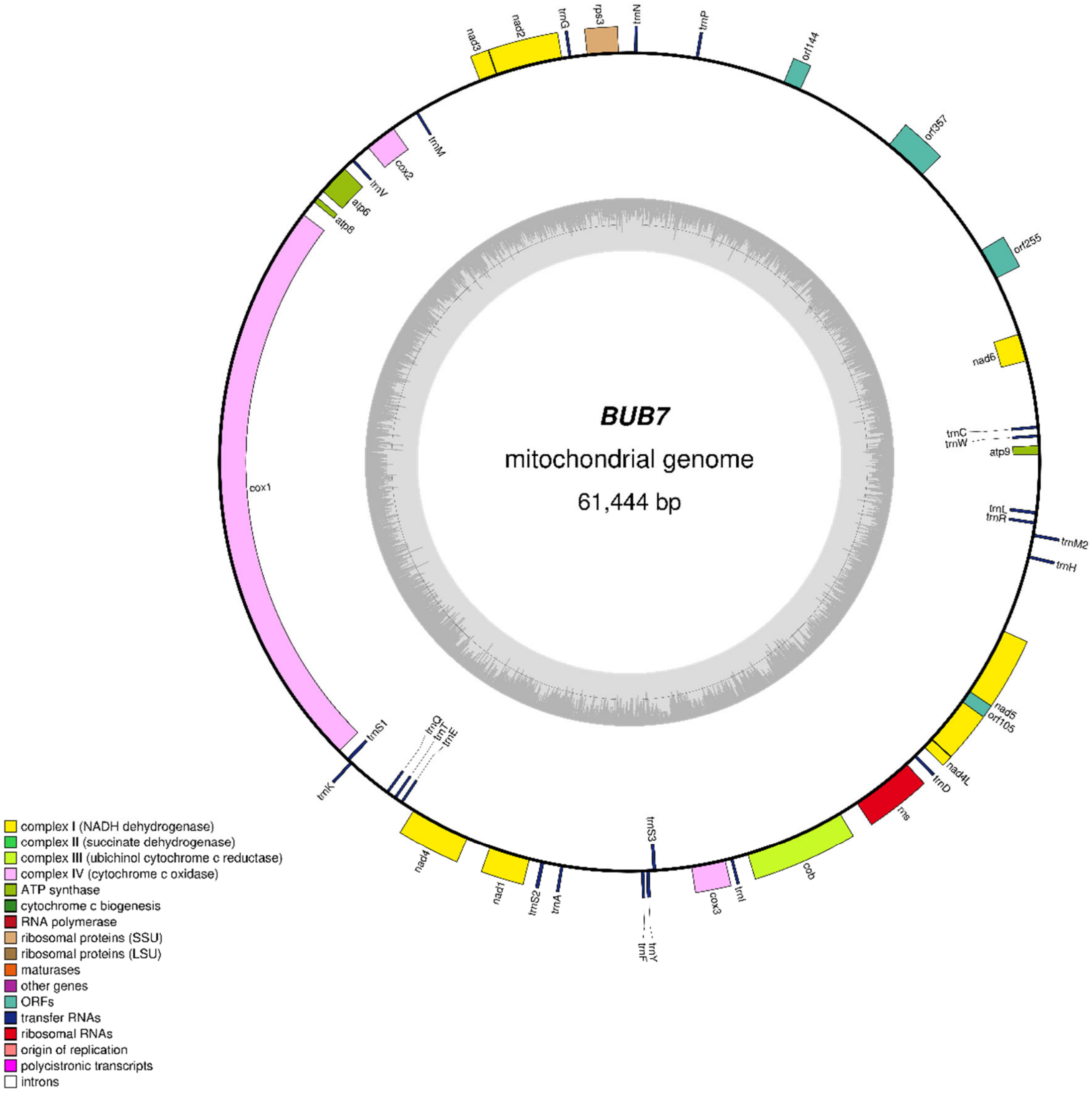

BUB7
mitochondrial genome
61,444 bp
nad3
nad2
trnG
rps3
trnN
trnP
orf144
orf357
orf255
nad6
trnC
trnW
atp9
trnL
trnR
trnM2
trnH
nad5
orf105
nad4L
trnD
rns
cob
trnI
cox3
trnY
trnF
trnS3
trnA
trnS2
nad1
nad4
trnK
trnS1
trnQ
trnT
trnE
cox1
atp8
atp6
trnV
cox2
trnM
complex I (NADH dehydrogenase)
complex II (succinate dehydrogenase)
complex III (ubichinol cytochrome c reductase)
complex IV (cytochrome c oxidase)
ATP synthase
cytochrome c biogenesis
RNA polymerase
ribosomal proteins (SSU)
ribosomal proteins (LSU)
maturases
other genes
ORFs
transfer RNAs
ribosomal RNAs
origin of replication
polycistronic transcripts
introns

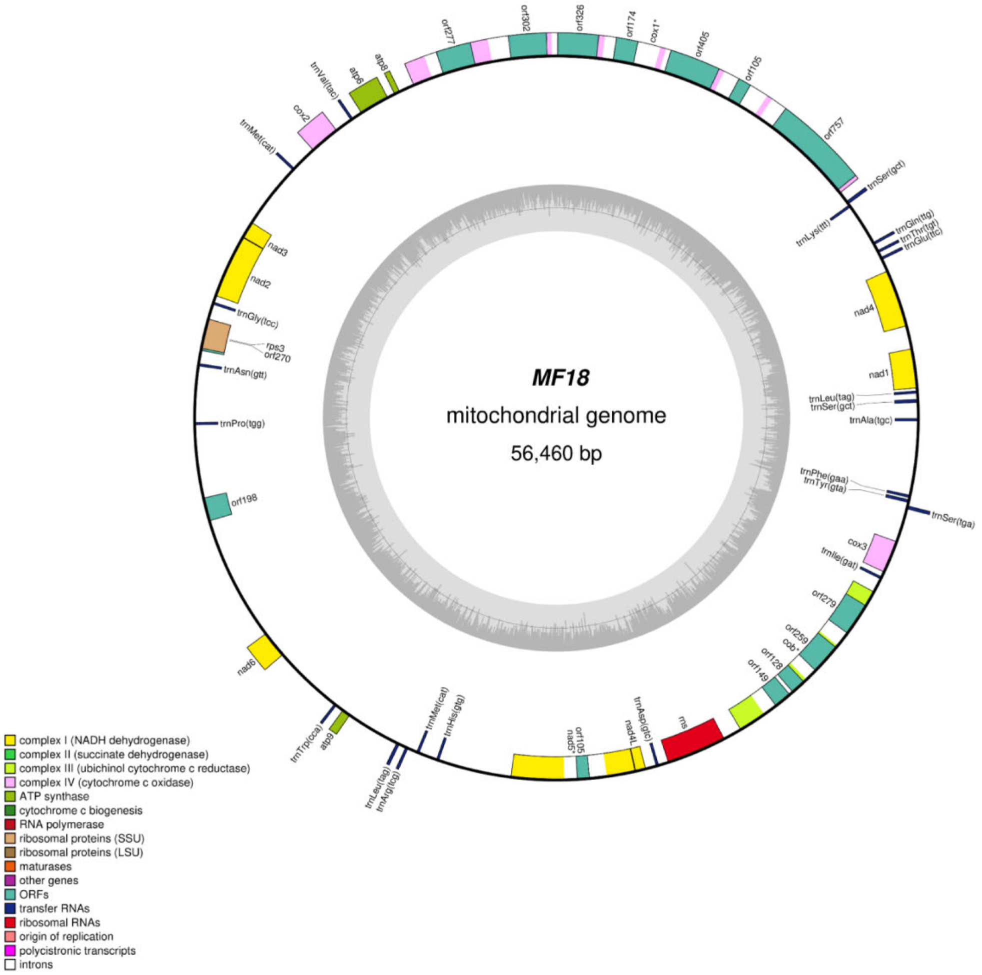

Figure S1. Circular maps of the mitogenomes of the different strains of *U. maydis*. Genes are represented by different colored blocks. Colored blocks outside each ring indicate that the genes are on the direct strand, while colored blocks within the ring indicate that the genes are

located on the reverse strand. Strains with the same genomes were represented as single maps. Map A represents strains 521, FB1, FB2, FB6A, FB6B and MF14, and map B represents GF5, GF8, GF25 and GF63 strains.

GENOME ORDER AMONG STRAINS.

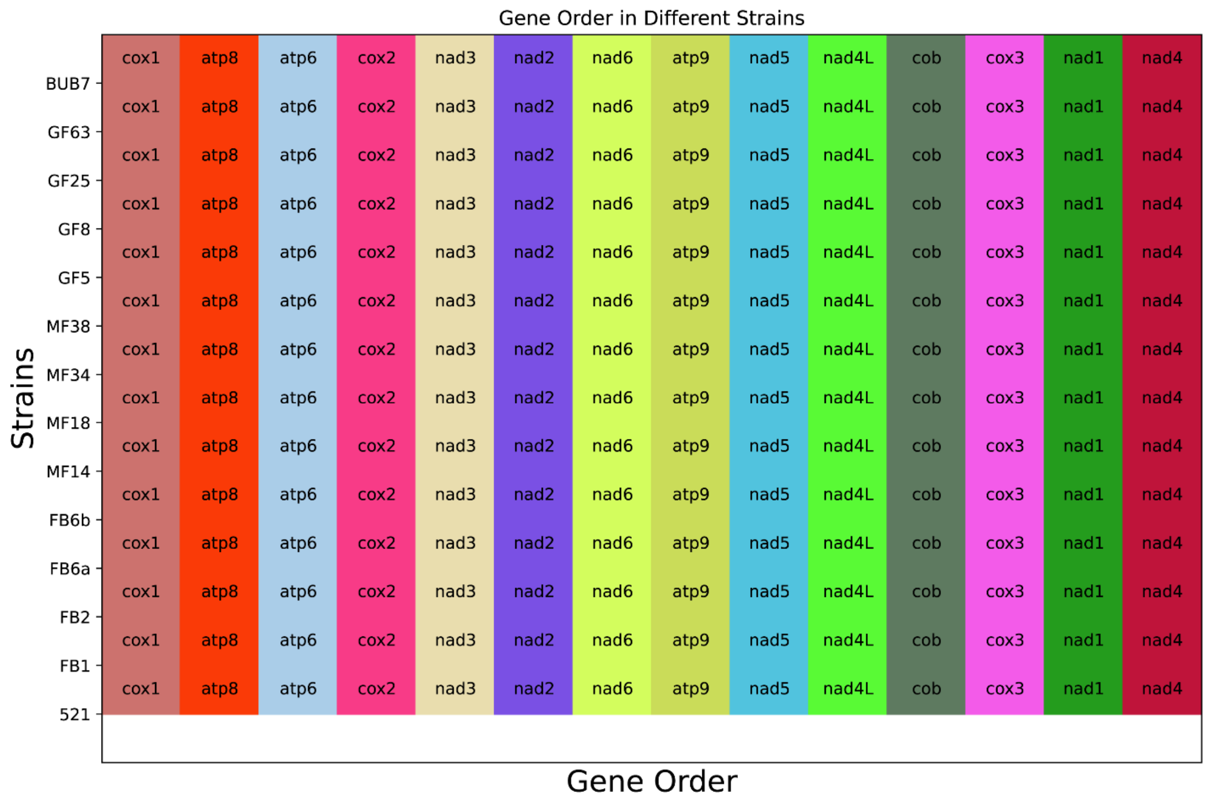

Figure S2. The gene synteny analysis across the 14 strains analyzed revealed that the 14 protein coding genes (PCGs) are highly conserved and maintained consistent gene order organizational structures throughout.

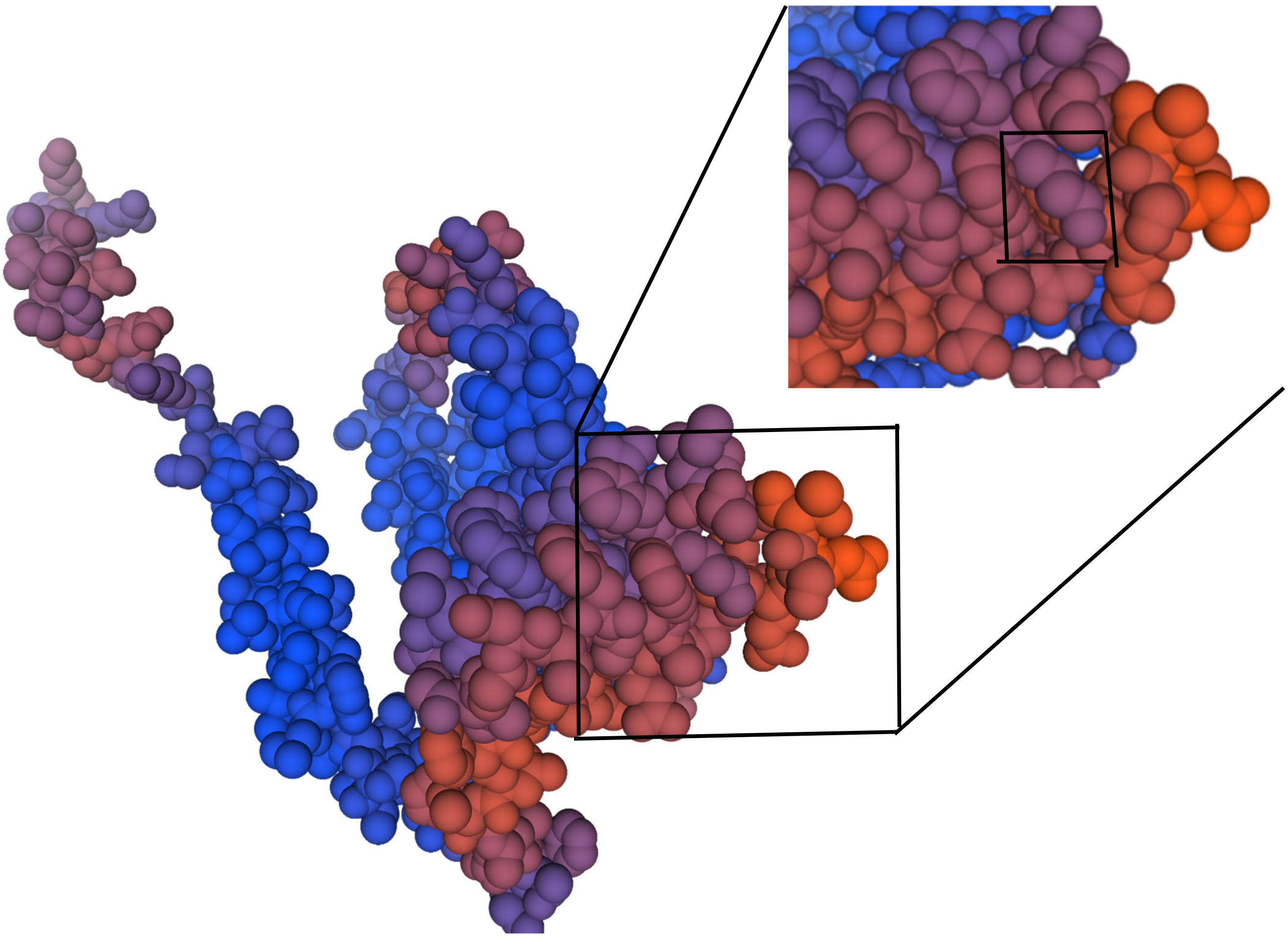

Figure S3. The protein structure in Nad6 is maintained in both sequences. The amino acid change from lysine (K) to glutamic acid (Q) had no influence on the protein structure. The predicted structure suggests the change happening on the outside of the protein, possibly not in the active site as highlighted. This may suggest an event where a different amino acid with different properties may replace another amino with no consequences. Protein structures were predicted by AlphaFold [27] (https://alphafoldserver.com/welcome).

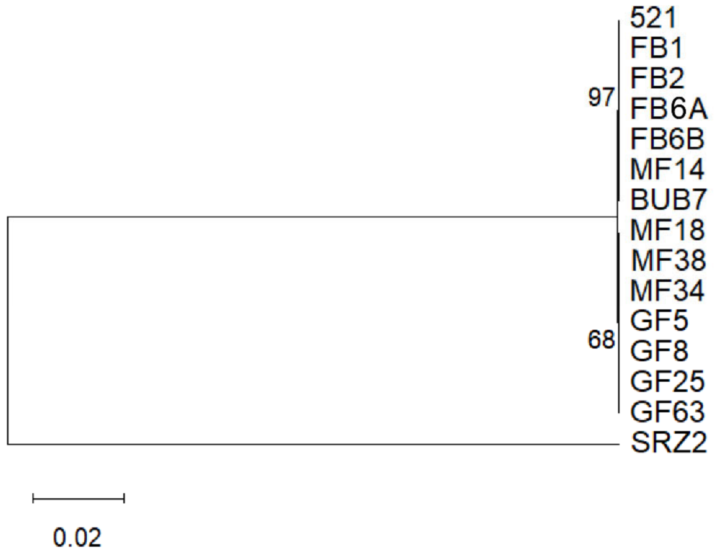

Figure S4. Phylogeny of mitogenomes in this study. Phylogenetic tree using the *Sporisorium reilianum* SRZ2 mitogenome [28] as an outgroup. The tree was calculated from multiple sequence alignment of the combined mitochondrial gene set (14 PCGs) of the 14 strains used. Phylogeny topology was inferred using the Tamura 3-parameter, with the maximum likelihood option. The phylogenetic tree was drawn using MEGA 12 software [25].

PRIMER SET 1

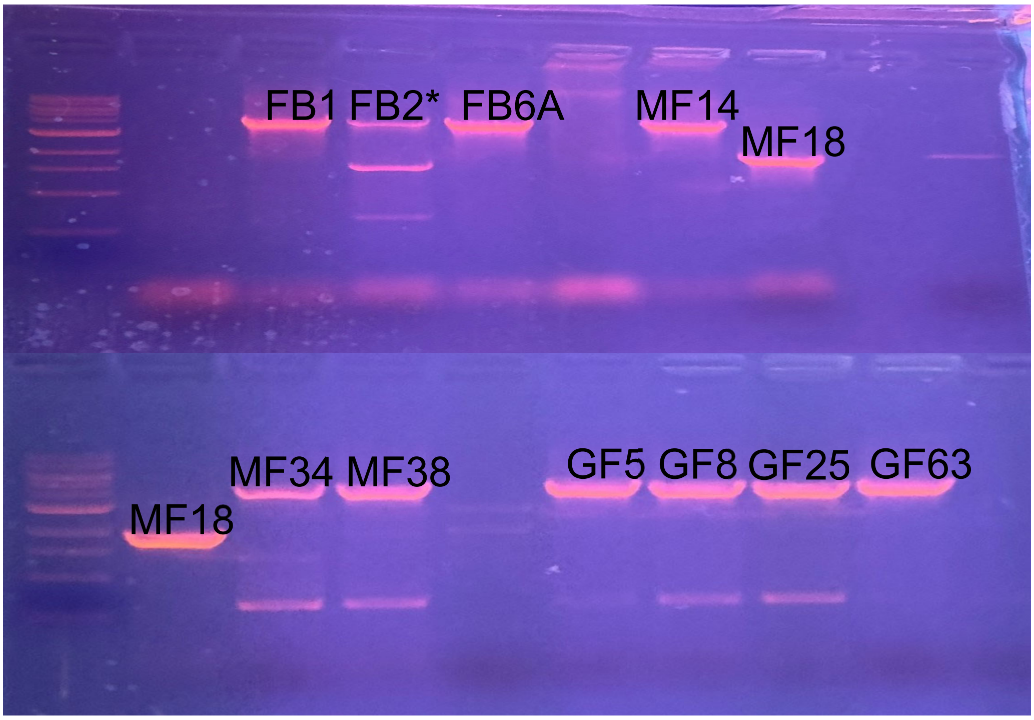

PRIMER SET 2

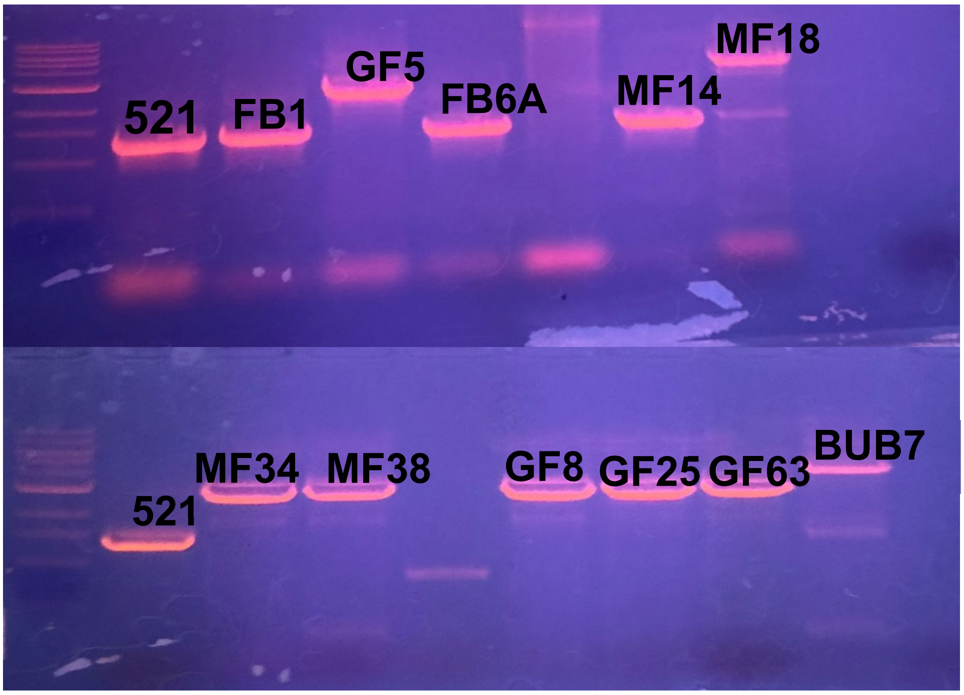

PRIMER SET 3

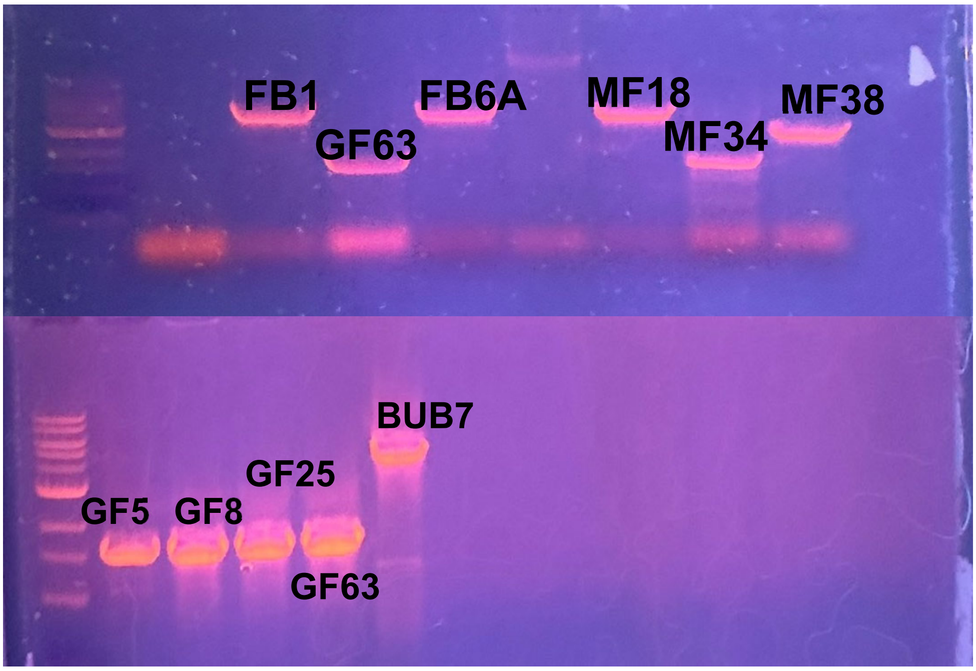

PRIMER SET 3

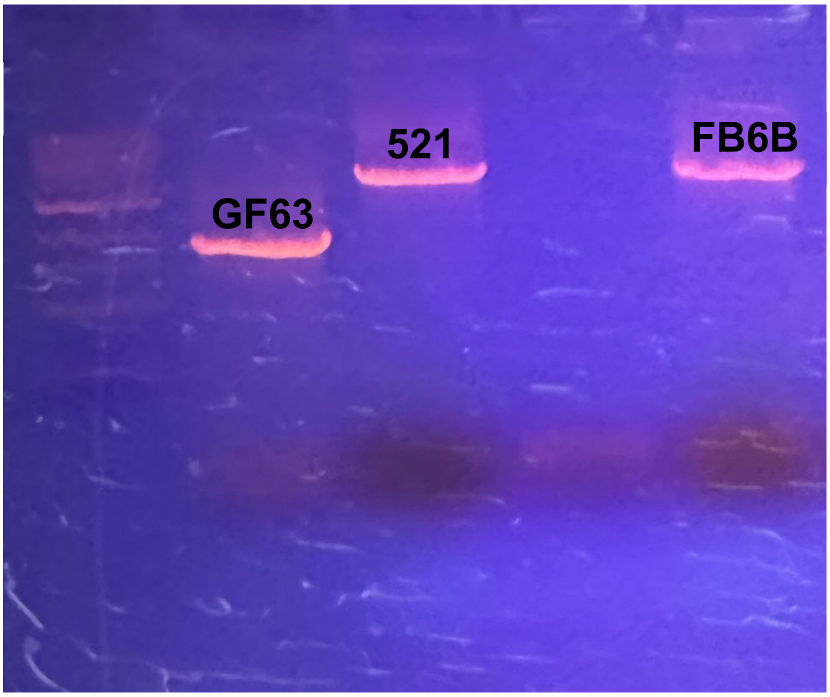

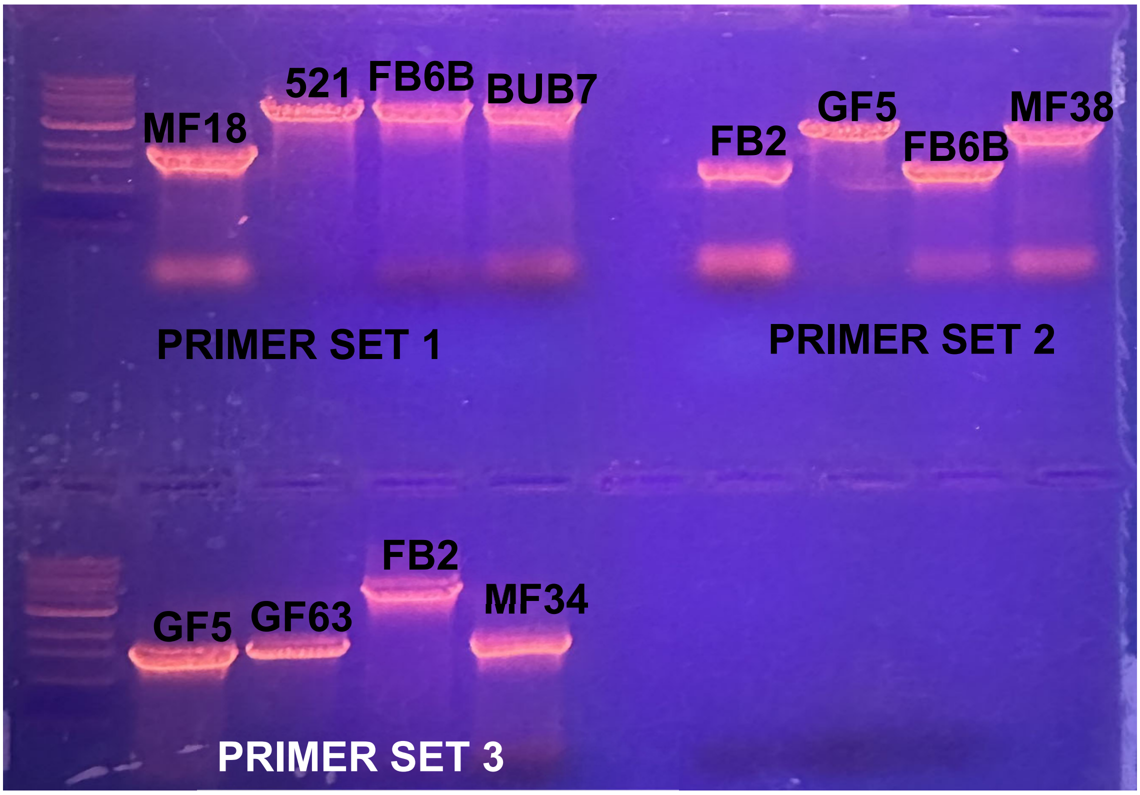

Figure S5. Gel images from mitotype determination. Figure 8 was generated from compilation of these data. Shown are the results of different primer sets (Primer Sets 1, 2 and 3) whose combinations were used to distinguish mitotypes of the 14 different *U. maydis* strains used in this study.
